# Sleep Apnea–Hypopnea Syndrome and Sleep Bruxism: A Systematic Review

**DOI:** 10.3390/jcm12030910

**Published:** 2023-01-23

**Authors:** Ana González González, Javier Montero, Cristina Gómez Polo

**Affiliations:** Department of Surgery, School of Medicine, University of Salamanca, 37007 Salamanca, Spain

**Keywords:** sleep bruxism, sleep apnea–hypopnea, systematic review

## Abstract

The aim of this study was o determine the relationship between sleep bruxism (SB) and sleep apnea–hypopnea syndrome (SAHS) at the pathophysiological level, the risk factors, as well as the common signs and symptoms. A search was carried out using the databases PubMed, Web of Science, Scopus, and the Cochrane Library together with the Boolean equation “bruxism” AND “sleep apnea” AND “relation*”; the systematic search strategy limited the results to English language articles published from 2013 until December 2021. This review was conducted in accordance with the PRISMA statement. Nine articles were reviewed to relate SAHS and SB at different levels: two were systematic reviews (22%) and seven were research studies (78%). According to the literature reviewed, SB and SAHS occur simultaneously in 21.0% to 41.3% of cases. There are signs and symptoms that are common to both SAHS and SB. Rhythmic masticatory muscle activity (RMMA) precedes an SAHS event in 25% of subjects with SB, in contrast to 55% of the general population. SB and SAHS seem to have a certain concomitance, ranging between 20% and 40%, and they also share some risk factors such as advanced age, obesity, smoking, and alcohol consumption. Dentists should be aware of this relationship, as part of a multidisciplinary team, for early diagnosis.

## 1. Introduction

Sleep-related disorders affect the architecture of sleep in its total duration, its onset, and/or maintenance, by affecting the quantity and/or quality of sleep [1]. Two of the main problems related to sleep and oral health are sleep apnea–hypopnea syndrome (SAHS) and sleep bruxism (SB) [2,3,4]. SAHS is a respiratory disorder in which breathing is intermittently and repeatedly interrupted during sleep, forcing the subject to expend respiratory effort to overcome transient decreases in oxygen saturation [5]. These episodes often lead to micro-awakenings that disrupt normal sleep patterns, causing excessive daytime sleepiness, general tiredness, nonrestorative sleep, and/or insomnia [5]. The most common signs and symptoms associated with SAHS are loud snoring, episodes of nocturnal choking, frequent awakenings accompanied by breath holding, gasping, or choking, nocturia, and/or morning headaches [6].

The global prevalence of SAHS was estimated in the analysis conducted by Benjafield et al. in 2019 [7]. After evaluating population-based studies from across the world, they concluded that 936 million adults aged between 30 and 69 (irrespective of sex) have mild to severe levels of SAHS globally, the country with the largest numbers of affected individuals being China, followed by the US, India, and Brazil. The authors stated that the use of different criteria could generate much higher figures for SAHS prevalence. This could lead to a potential underestimation of global obstructive sleep apnea prevalence (of up to −48%). Prevalence exceeds 50% in countries such as Brazil, Germany, Singapore, Switzerland, and Japan, with the study providing specific data for each nation [7].

The prevalence of SAHS increases with age, and, in children, the syndrome has a prevalence between 0.2% and 4.1% that peaks between the ages of 2 and 6 years [5,6]. The most notable risk factors associated with SAHS are age, sex (males being at more risk), and a high body mass index [8,9]. Other factors include the use of alcohol, tobacco, or drugs, genetic and racial factors, and the supine position during sleep [10,11]. As a sleep disorder, SAHS has been studied using polysomnography (PSG) [8,12], which measures the sleep apnea–hypopnea index (AHI) and the oxygen desaturation index (ODI) [5,12]. Through these measurements, the pathology is classified as mild if there are 5–14 events per hour, but moderate for 15–30 events, and severe for more than 30 events per hour, while hypoxemia is considered to be a decrease in blood oxygen of 90% or less [5]. PSG is the gold-standard diagnostic method of SAHS, but it is a high-cost test due to the number of personnel involved, the equipment required, the high level of technical training needed, and the overnight stay in hospital [5,12,13]. In recent years, to reduce waiting lists and associated hospital costs, simpler diagnostic methods based on clinical suspicion that help to reach an earlier diagnosis have been used, such as questionnaires and portable overnight respiratory polygraphy.

Sleep bruxism [14,15] occurs during sleep (it does not necessarily occur at night). According to Lobbezoo et al., SB can be defined as follows: “Sleep bruxism is a masticatory muscle activity during sleep that is characterized as rhythmic (phasic) or nonrhythmic (tonic) and is not a movement disorder or a sleep disorder in otherwise healthy individuals”. The phrase “otherwise healthy” indicates that bruxism is not a disorder if the patient does not have a concomitant disorder, such as a sleep disorder like SAHS or a movement disorder like epilepsy. If the patient is suffering from such a condition, SB would be a sign of its presence [16].

This activity can lead to tooth wear [11,16,17,18], tooth migration [11,18], occlusal trauma [11,19,20,21,22], muscle pain [14,16,17,18], temporomandibular disorders [11], and headaches [11,16,17,18]. The prevalence of SB is estimated at 15–23% in the general population, with 14% of children (especially during the mixed dentition stage), 8% of adults (mainly between 19 and 45 years of age), and 3% of elderly people affected [11,15,19,20,23]. Most of the percentages were obtained from questionnaires and not from instrumental data. Most authors argue that there is no difference between the sexes [20,21]. Several risk factors for SB have been described, including obesity, [11,18,24,25] smoking [11,23,25,26], alcohol use [11,23,25,26], high coffee consumption [25,26], drugs such as amphetamine [25], oral habits such as onychophagia [25,26], and drugs such as serotonin receptor inhibitors [26], calcium blockers [23,25,26], antiarrhythmics [25,26], antidopaminergics [23,25,26], and antipsychotics [23,25,26]. SB is also associated with psychological factors [11,27], stress [11,27], depression [11,27], and emotional instability (neuroticism) [11,27], which influence the functions of the autonomic nervous system [16]. Factors such as snoring, restless sleep, night drooling, sleeping position, and lack of sleep may trigger the onset of SB [11,18,24,25]. SB is a risk factor or a protective factor, according to Lobbezoo et al. (2018) [16]. By recognizing SB as a protective factor in patients with SAHS, early diagnosis in patients with SB by dentists is important to explore a possible SAHS. The diagnosis of SB is classified as (1) possible when it is based on a self-survey (through a questionnaire) and/or clinical examination, (2) probable when the self-survey is combined with an intraoral clinical examination (wear, facets, and erosion), and (3) definite if SB is present in the self-survey and in the clinical examination, and if there is a polysomnographic record with a bruxism episode index (BEI), which takes into account RMMAs related to clenching of the teeth per hour [27]. The BEI indicates that there is no bruxism if RMMA events per hour are less than two, that bruxism activity exists but is low if more than two and up to four RMMA events occur per hour, and that SB exists and has a high activity if there are more than four RMMAs per hour during sleep [27]

**Figure 1:**
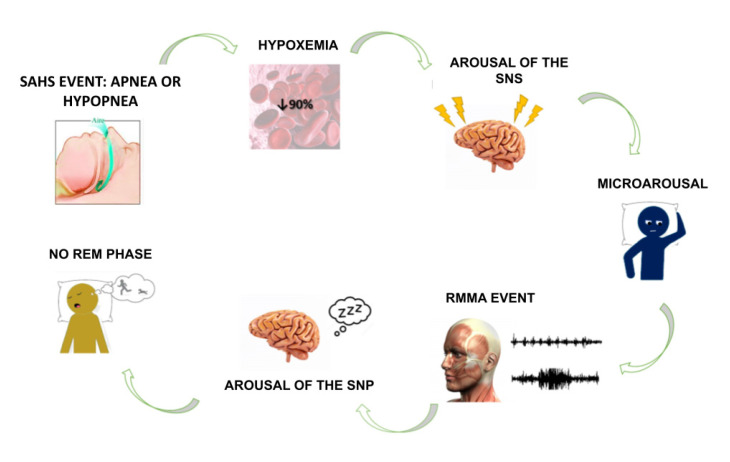
Diagram showing the possible mechanism linking SAHS and SB.

Regarding the relationship between SAHS and SB, there are two hypotheses: (1) RMMA may be an oral motor activity that helps to restore airway patency after an obstructive breathing event during sleep [11,14,28]; (2) RMMAs are physiological motor events that are required to lubricate the oropharyngeal structures during sleep—as salivary flow and the swallowing rate are reduced—and to reposition the jaw [19,23,28]. It has not been established whether SAHS causes RMMA or the other way around [15,19,20]. However, research has found that RMMA precedes an SAHS event in 55% of cases [19], while, in 25% of SB patients, an apnea or hypopnea event precedes RMMA [20] (Figure 1).

Defining the mechanisms of onset and the causal relationship between SAHS and SB still requires further research [11,14,19,23], but a study carried out in Poland associated SB and SAHS in different population samples, finding that they are concomitant [28]. Martynowicz et al. found a 50% co-prevalence in a sample of patients with SAHS, where the disorder was associated with moderate SB [28]. These findings point to a possible link between SB and SAHS. PSG would be the ideal diagnostic method to confirm this potential relationship between SB and SAHS, but its complexity and high cost necessitate optimizing its use [12,13]. Therefore, questionnaires have been developed to serve as a diagnostic filter [6]. For SAHS diagnosis, the data recommended to collect include the presence of excessive daytime sleepiness [8,28], snoring, tiredness, patient observations, blood pressure, body mass index, age, neck circumference, and gender, whereas questions on night-time grinding sounds, micro-awakenings, and daytime muscle tension are used to screen for SB [8,28]. Nevertheless, the use of questionnaires as a screening method often leads to the number of SB and SAHS cases being underestimated [5]. Therefore, in recent years, there has been a radical increase in the use of portable recording devices to diagnose SAHS. These portable devices perform home respiratory polygraphy or nonhospital registration; the most widely used are type IV and consist of three recording channels (respiratory effort recording, air flow, and pulse oximeter) [5,12,27]. PSG, however, records up to eight channels: electromyography, electroencephalography, electrooculography, nasobuccal breathing (registers AHI), abdominal breathing (differentiates obstructive events from central ones), leg movements, heart rate in beats per minute, oxygen saturation in blood, and body position [12]. During the PSG, the BEI can be determined if one more recording channel is added, i.e., electromyography of the mandibular elevator muscles, usually the masseters [5,12,27]. Portable respiratory polygraphy has a high correlation with PSG results in uncomplicated cases of SAHS, making testing less expensive. In summary, if the relationship between SB and SAHS is better understood, diagnosis and treatment could be more effective in improving quality of life and preventing nonrestorative sleep [14,22]

This literature review aims to assess the state of the art on (1) the relationship between SB and SAHS, and (2) the signs and symptoms common to SB and SAHS, so as to identify a prototype at-risk patient.

## 2. Materials and Methods

This review was conducted in accordance with the PRISMA statement (www.prisma-statement.org), and the authors followed the PROSPERO 2016 CRD42016043324 protocol.

The PICO question for this review was as follows: “Is there a relationship between SB and SAHS?”

- Patient: a person with SB and concomitant SAHS;
- Intervention: pathophysiological relationships and common clinical picture according to common diagnostic methods (self-survey, examination, and PSG);
- Comparison: signs/symptoms and differential findings;
- Outcome: improved diagnostic efficiency of SB and SAHS;

For this review, the PubMed, Web of Science, Scopus, and Cochrane Library databases were selected as information and search sources, using the descriptors “bruxism”, “sleep apnea”, and “relation” with the Boolean operator “AND”. However, the articles used for the introduction were obtained from other searches. As inclusion criteria, the studies had to be either a systematic review or research conducted on humans, analyze SB and SAHS together, and have been published between 2013 and 31 December 2021. The data extracted from each study were organized into three tables, according to the type of study and the subject matter: systematic reviews linking SB and SAHS (Table 1); clinical trials analyzing pathophysiological relationships between SB and SAHS (Table 2); research studying the signs and symptoms associated with SB and SAHS (Table 3). Risk of bias of clinical trials was detected and classified using RevMan 5 (Review Manager (RevMan) [Software]). Version 5.3. Copenhagen: The Nordic Cochrane Center, The Cochrane Collaboration, 2020).

## 3. Results

**Figure 2:**
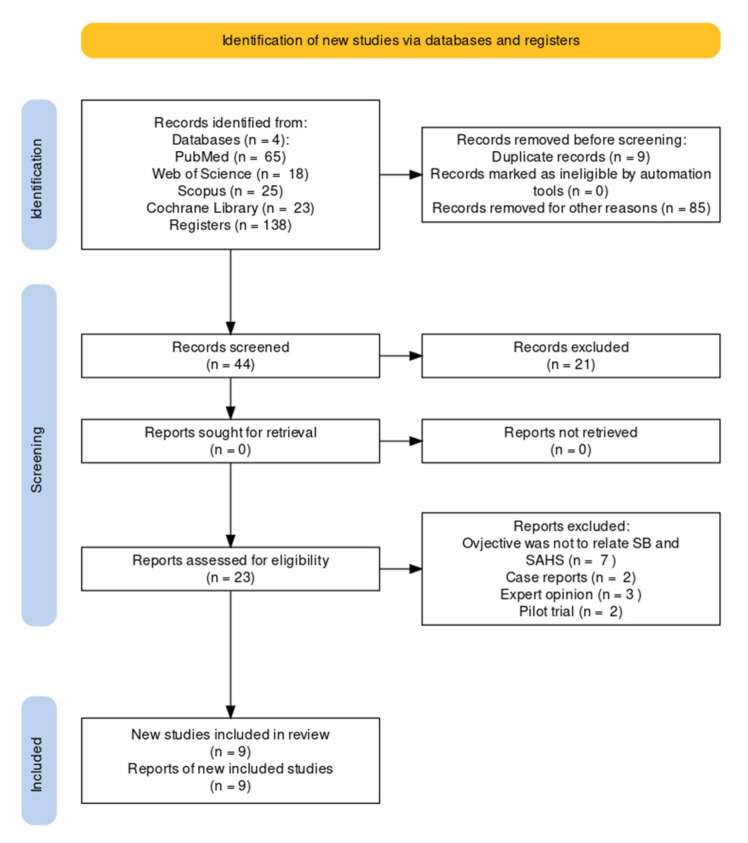
Flowchart outlining the literature search.

From the literature search, a total of 138 articles were retrieved, of which 53 were selected. After eliminating duplicates, only 44 articles remained; however, 18 were eliminated according to the title and abstract. Thus, a total of 26 full-text articles were reviewed. Finally, nine articles were consulted that fulfilled the inclusion criteria on the concomitance of SB and SAHS. The strategy for conducting the literature search is detailed in the flowchart in Figure 2.

In the tables, information was collected on the authors and the year of publication, creating a timeframe ranging from 2013 to 2021. The types of studies analyzed were as follows: two systematic literature reviews (22%) [29,30] and seven research papers (78%) [31,32,33,34,35,36,37] (of which four were clinical studies (45%) [31,32,34,36], two were retrospective studies (22%) [33,35], and one was a cross-sectional study (11%) [37]). The column summarizing the objectives of each study was essential for sorting the papers according to their subject matter, resulting in three groups: systematic literature reviews on the relationships between SB and SAHS (Table 1), pathophysiological relationships between SB and SAHS (Table 2), and signs and symptoms associated with SB and SAHS (Table 3). The method used to identify relevant articles was consulting the references cited in the reviews, and determining whether questionnaires were completed [36,37] and/or PSG and EMG recordings used.

Systematic reviews linking SB and SAHS (Table 1). The systematic review by Jokubauskas and Baltrušaitytė argued that more trials are needed to explain why SB occurs after a micro-awakening, following an SAHS event with hypoxemia [29]. Their synthesis [29] included clinical trials (which are not retrospective or cross-sectional) published between January and September 2016. The most up-to-date systematic review was that authored by Da Costa Lopes et al. [30] in which seven articles were studied in detail. The authors conclude that there is insufficient evidence to support the relationship between SB and SAHS and that more studies are needed. Their review included studies published up to May 2019. Both systematic reviews (Da Costa et al. [30] and Jokubauskas et al. [29]) focused solely on the adult population and on patients diagnosed with SAHS using PSG.

Clinical trials examining pathophysiological relationships between SB and SAHS (Table 2). In the trial by Hosoya et al. [31] in 2014, 67 patients with SAHS and 16 healthy patients underwent PSG recordings to determine the risk of SB. On the basis of this study, the authors concluded that patients with SAHS show a higher risk of eccentric SB. Two years later, Saito et al. [32] carried out a study using PSG on 59 participants and concluded that SAHS is related to nonrhythmic SB, but RMMA is not related to awakenings. In 2020, Suzuki et al. [35] monitored 12 patients by PSG and showed that there is a drop in O_2_ saturation before RMMA; therefore, they contended that SB and SAHS are related. Tan et al. [33] analyzed 147 PSGs to establish that one-third of the sample suffered from SB and SAHS simultaneously, and that RMMA is a protective phenomenon against SAHS-derived hypoxemia. Smardz et al. [34] concluded that centric SB is associated with SAHS. All previous studies agreed that SB and SAHS are connected by the mechanism through which they are produced; however, there are variations in the categories of events found, and more research is still needed.

Research into the signs and symptoms associated with SB and SAHS (Table 3). In 2016, Tachibana et al. [36] analyzed questionnaires from 6023 parents. According to this study, it appears that 21% of the sample of children examined presented SB that was related to SAHS, due to common symptoms such as nocturnal movement, oral breathing, the head being in a backward position, snoring, apnea–hypopnea, and gasping. Five years later, Laganà et al. [37] assessed 310 questionnaires, according to which 41.3% of the subjects had SB accompanied by SAHS risk factors such as heredity, night sweats, nocturia, oral breathing, and snoring. Both papers concluded that there are risk factors, signs, and symptoms shared by SB and SAHS.

## 4. Discussion

In the nine articles reviewed, the relationship between SB and SAHS was examined with different objectives in mind: to try to understand the pathophysiological mechanism linking SAHS and SB, and to identify the shared signs and symptoms as a means to develop a generalized model that may facilitate diagnoses.

The studies discussed can be considered current, as they were all carried out less than 10 years ago. The only paper included in the study presenting both a control and a study group is that by Hosoya et al. [31] as the other articles can be considered clinical studies. Most authors used the PSG diagnostic test, which is the gold standard for diagnosing SAHS and SB, and for determining physiological relationships [31,32,33,34,35]. Only Laganà et al. [37] and Tachibana et al. [36] surveyed parents, as their research was carried out on a pediatric population, and the test employed could be too invasive for children. Systematic reviews included a minimum of three [29] and a maximum of seven articles [30], and the adult clinical studies that used PSG as a method to compare outcomes had samples with a maximum of 147 [33] and a minimum of 12 patients [35], although the follow-up was only for one night [31,32,33,34,35]. The pediatric studies that conducted clinical examinations and parental questionnaires involved samples of 310 children in the case of Laganà et al. [37] and 6023 in the research by Tachibana et al. [36].

Not all studies had “standardized” criteria, and diagnostic criteria differed between studies. This is a limitation when it comes to establishing an action algorithm in the face of a possible relationship between SB and SAHS.

Systematic reviews relating SB and SAHS (Table 1). The two systematic reviews agreed that the role of the dentist in SB diagnosis and preventive detection of SAHS is very important [29,30]. One of these reviews contended that there is a relationship between SB and SAHS but did not confirm it, as it was poorly supported by the available scientific literature [29]. In contrast, the systematic review by Da Costa Lopes et al. did not establish a link between SB and SAHS, due to a lack of scientific evidence [30]. While there may be links in the production of SB and SAHS, these have not been scientifically proven; hence, more studies are needed to validate this association. For these literature reviews to be more conclusive, clinical research would need to study larger sample sizes that are representative of the population, carry out appropriate follow-ups, and examine all possible variables related to SAHS and SB. The present systematic review differs from those described above [29,30] in that it includes publications dated up to December 2021, broadens the bibliographic sources by including Scopus, covers the pediatric population, and not only draws on research using the PSG methodology but also covers that using portable overnight respiratory polygraphy and validated questionnaires. According to Lobbezoo et al. [16], there are indicators of possible/probable bruxism for which a PSG is not necessary.

Clinical research analyzing pathophysiological relationships between SB and SAHS (Table 2). All the clinical studies established a relationship between SB and SAHS; however, there were discrepancies when it came to identifying a specific type of muscular movement of SB, as some studies related SAHS to phasic SB [31] and others to tonic SB [34]. They also agreed that SAHS produces micro-awakenings due to hypoxemia (cause), and that SB is not responsible but is derived from them (consequence) [31,32,33,34,35]. The literature holds that SAHS involves hypoxemia and that SB and micro-arousals occur in response to this oxygen depletion, but independently of each other [29,31,32,33,35]. The specific type of muscular movement of bruxism related to SAHS still needs more analysis. Bruxism related to SAHS still needs further analysis, since, in many cases, there are RMMA events associated with SAHS events, either before or after, but they are not enough throughout the hours of sleep to diagnose bruxism (BEI < 2). In addition, research would be more informative if it were longitudinal, with a study group and a control group. Lastly, representative SAHS parameters should be recorded by PSG, and SB parameters should be recorded by EMG.

Research into the signs and symptoms associated with SB and SAHS (Table 3). In the pediatric studies, it was established that between 21% [36] and 41.3% [37] of the children had SB and SAHS simultaneously. They were related by a similar clinical picture, consisting of nocturnal movement, mouth breathing, the head being in a backward position, snoring, apnea–hypopnea, and gasping [36], and common risk factors including heredity, night sweats, nocturia, mouth breathing, and snoring seemed to correlate with bruxism [37]. According to the clinical guidelines of the American Sleep Medicine Association [28], the prevalence of patients with SB and concomitant SAHS in the pediatric population is significant but thought to be underdiagnosed.

Self-reports or questionnaires are the main tool to assess SB [16]. This method can be useful for suspected bruxism; however, for more complex cases, dental professionals need to have more clinically relevant diagnostic devices. Given the possible link between SB and SAHS, the dentist has an important role in screening patients, as well as in the detection of certain risk factors. To meet this goal, dentists should have basic training in sleep medicine. On many occasions, they do not suspect the presence of SAHS due to ignorance and neither treat it themselves nor refer their patients to otolaryngologists or pulmonologists. Furthermore, patients with suspected SAHS can be screened in dental clinics by means of portable respiratory polygraphy records. Under these conditions, diagnosis would be faster, and referral for multidisciplinary treatment would be more efficient. With adequate training in dental sleep medicine, treatment with mandibular advancement devices could improve the patient’s quality of life and sleep.

## 5. Conclusions

1. SB and SAHS seem to have a certain concomitance, ranging between 20% and 40%. The most widespread causal relationship suggests that hypoxemia resulting from SAHS causes a micro-awakening, and the RMMA in SB is generated to protect the sleep cycle from a drop in the level of oxygen. To corroborate this conclusion, more quality literature is needed that deals with the relationship between SB and SAHS.
2. The prototypical at-risk patient appears to be an obese adult man of advanced age, and the main shared signs and symptoms are poor sleep quality, mouth breathing, nocturnal snoring, a preference for a supine sleeping position, and morning headaches. The dentist has a fundamental role in screening but has to work in an interdisciplinary team since very few dentists have sufficient training in dental sleep medicine.

## Figures and Tables

**Table 1:** Systematic reviews linking SB and SAHS.

Author(s) and Year	Type of Study	Objective	M&M	Parameters	Conclusions
Jokubauskas and Baltrušaitytė 2017 [29]	Systematic review	Linking SB and SAHS	Bibliography	3 articles	Further studies are needed to understand why bruxism occurs after a micro-awakening following an SAHS event with hypoxemia.
Da Costa Lopes 2020 et al. [30]	Systematic review	Relationship between SAHS and SB	Bibliography	7 articles	There is no scientific evidence to support a conclusive link between SB and SAHS. More studies are needed.

M&M: materials and methods.

**Table 2:** Clinical studies analyzing pathophysiological relationships between SB and SAHS.

Authors and Year	Type of Study	Objective	M&M	Parameters	Risk of Bias	Conclusions
Hosoya et al. 2014 [31]	CS	Linking SB, SAHS, and awakenings	PSG on 67 SAHS patients and 16 healthy patients (control group) for 1 night	Electroencephalogram, electrooculogram, electromyogram, respiratory flow, snoring, respiratory movements of the abdomen, saturation of oxygen, and electrocardiogram	Moderate (selection bias)	Patients with SAHS are at high risk of SB. This is the first report linking phasic type SB (eccentric) with obstructive apnea events. Improvement of SAHS could prevent exacerbation of SB.
Saito et al. 2016 [32]	CS	Linking apnea and bruxism	PSG and recording of 59 participants for 1 night	RMMA and awakenings	Moderate (attrition bias)	There is no association between RMMA and awakenings, most movements during apnea are non-rhythmic, and SAHS and SB are related by some type of mechanism.
Tan et al. 2019 [33]	Retrospective CS	Determine the prevalence of SB in patients with SAHS	PSG on 147 patients diagnosed with SB (SBI > 4) for 1 night	Micro-awakenings, DOI, AHI	High (selection bias)	One-third of the patients had both SB and SAHS. They presented more micro-arousals; therefore, RMMA may be related as a protective phenomenon against hypoxemia-related micro-arousals.
Smardz et al. 2020 [34]	CS	SB and SAHS relationship	77 patients with suspected SB undergoing PSG and EMG for 1 night	BEI, the type of RMMA, AHI and DOI	High (selection bias, performance bias)	Tonic contractions are related to SAHS events.
Suzuki et al. 2020 [35]	Retrospective CS	Relationship between SB and fluctuation of O_2_ and CO_2_	PSG and EMG for 1 night on 12 patients	RMMA, changes in O_2_ saturation, exhaled CO_2_, and apnea and hypopnea events	High (selection bias)	Before an RMMA, the O_2_ saturation drops, but this does not affect the CO_2_ expelled. The relationship between SAHS and SB is physiological.

M&M: materials and methods; CS: clinical study.

**Table 3:** Clinical research into the signs and symptoms associated with SB and SAHS.

Authors and Year	Type of Study	Objective	M&M	Parameters	Risk of Bias	Conclusions
Tachibana et al. 2016 [36]	CS	To examine the prevalence of sleep bruxism in children in Japan, and its connections to sleep-related factors and daytime problematic behavior	Japanese Sleep Questionnaire for parents and caregivers of 6023 children aged 2–12 years	SAHS, restless legs syndrome, morning symptoms, sleep habits, parasomnias, insufficient sleep, excessive daytime sleepiness, daytime behavior, and insomnia or circadian rhythm disorders.	Low	In total, 21% of children have SB. SB is related to obstructive SAHS through the following symptomatology: night movement, mouth breathing, the head being in a backward position, snoring, apnea–hypopnea, and wheezing.
Laganà et al. 2021 [37]	Cross-sectional CS	Relation between SB and SAHS risk factors	Questionnaires for both parents of 310 children	Personal data, sleep quality, and risk factors for SAHS	Low	In total, 41.3% of the sample had SB and 46.5% of the parents did not know what SB was. Risk factors for SAHS—such as heredity, night sweats, nocturia, mouth breathing, and snoring—seem to correlate with bruxism.

M&M: materials and methods; CS: clinical study.

## Data Availability

The data will be available upon a reasoned request.
